# A Fast Binocular Localisation Method for AUV Docking

**DOI:** 10.3390/s19071735

**Published:** 2019-04-11

**Authors:** Lijia Zhong, Dejun Li, Mingwei Lin, Ri Lin, Canjun Yang

**Affiliations:** 1 State Key Laboratory of Fluid Power and Mechatronic Systems, Zhejiang University, Hangzhou 310027, China; zhonglijia@zju.edu.cn (L.Z.); mwlin_1023@163.com (M.L.); 21625088@zju.edu.cn (R.L.); ycj@zju.edu.cn (C.Y.); 2 Pilot National Laboratory for Marine Science and Technology (Qingdao), Qingdao 266000, China

**Keywords:** autonomous underwater vehicles, docking guidance technology, binocular vision

## Abstract

Docking technology plays a critical role in realising the long-time operation of autonomous underwater vehicles (AUVs). In this study, a binocular localisation method for AUV docking is presented. An adaptively weighted OTSU method is developed for feature extraction. The foreground object is extracted precisely without mixing or missing lamps, which is independent of the position of the AUV relative to the station. Moreover, this extraction process is more precise compared to other segmentation methods with a low computational load. The mass centre of each lamp on the binary image is used as matching feature for binocular vision. Using this fast feature matching method, the operation frequency of the binocular localisation method exceeds 10 Hz. Meanwhile, a relative pose estimation method is suggested for instances when the two cameras cannot capture all the lamps. The localisation accuracy of the distance in the heading direction as measured by the proposed binocular vision algorithm was tested at fixed points underwater. A simulation experiment using a ship model has been conducted in a laboratory pool to evaluate the feasibility of the algorithm. The test result demonstrates that the average localisation error is approximately 5 cm and the average relative location error is approximately 2% in the range of 3.6 m. As such, the ship model was successfully guided to the docking station for different lateral deviations.

## 1. Introduction

In recent decades, autonomous underwater vehicles (AUVs) have played an increasingly crucial role in marine exploration and development, such as resource detection, military technologies, and underwater structure inspections [1]. To facilitate the flexibility of their movement, the power capacity of AUVs is limited by their compact outfit. In addition, upon completion of undersea assignments, AUVs have to return to the bank to obtain a new mission. Docking technology makes it possible for the vehicles to upload data, download new assignments and recharge their batteries underwater [2,3]. This greatly extends the duration of their operation. To achieve this expected manipulation, AUVs must be guided to the docking station during the first step. The guiding process is divided into two stages: the homing stage and the docking stage [4]. AUVs are guided to the neighbourhood of the docking station during the homing stage and then enter into it during the docking stage. Compared with the homing stage, the docking stage requires higher localisation precision and directly determines whether AUVs can get back to the docking station successfully or not. As such, the docking technology for the docking stage is of vital importance.

In previous works, several types of docking technologies based on the use of different sensors have been introduced.

A combination navigation system including an ultra-short baseline (USBL) acoustic array and a Doppler velocity log (DVL) was proposed by Allen et al. [5] for REMUS AUV docking. McEwen et al. [4] developed a docking system for a 54-centimetre diameter AUV using USBL guidance technology near the docking station. Vallicrosa et al. [6] also used USBL technology to guide Girona500 I-AUV (Intervention AUV) to a docking station.

An electromagnetic (EM) docking system was first proposed by Feezor et al. [7] for the Odyssey AUV. In recent years, Vandavasi et al. [8] from the India National Institute of Ocean Technology described the concept and test of an electromagnetic homing guidance system (EMHGS). Peng, S. et al. [9] from Hangzhou Dianzi University and Zhejiang Provincial Key Lab of Equipment Electronics also developed a low-cost electromagnetic docking guidance (EMDG) system for micro AUVs.

Acoustic signals are easily disturbed by reflecting surfaces such as the seabed and target structures, which makes it hard to achieve high accuracy in a short operating range [10]. Electromagnetic signals have a high attenuation velocity underwater and can only be valid within a relatively short range [8,9]. In recent years, with the gradual improvement in computational capacity, vision guidance technology has been developed as a commonly used approach for AUV terminal docking due to its simplicity and effectiveness. It has excellent performance in clear water and can be effective within a range of 0–15 m [11].

Park et al. [12] proposed a vision docking system for ISIMI AUV with lights equipped at the entrance of the docking station and cameras equipped at the head of the AUV. By averaging the mass centres of the lights, AUV can obtain the relative two-dimensional position to the docking station and move towards its target. Their experiment was conducted in an ocean engineering basin (OEB) of the Korea Ocean Research and Development Institute (KORDI).

Y. Li et al. [13] built a vision docking algorithm that was a combination of monocular and binocular positioning methods. The algorithm switches between the two operating modes depending on the number of lights in the images captured by the two cameras and can obtain the six-dimensional pose of the AUV. However, the computational burden of this vision-based navigation is heavy, corresponding to approximately 1.5–2.5 s, and a dead reckoning algorithm is used for aided navigation. The experiment was conducted in the water pool lab at Harbin Engineering University.

D. Li et al. [11] presented a vision docking method using one camera and one light. The four stages taken to enable the AUV to obtain its relative distance to the docking station and navigate to the target can be outlined as: image acquisition, binarisation of the captured images, elimination of noisy luminaries, and estimation of the relative position. This system was tested in the swimming pool of Yuquan Campus of Zhejiang University.

N. Palomeras et al. [14] proposed a range-only localisation algorithm to approach the docking station and developed an associated estimation algorithm using active beacons and augmented reality (AR) markers to complete the docking manoeuvre at short ranges. When the camera on the AUV can capture all the lights at the docking station, the six-dimensional pose of the AUV can be estimated using a non-linear least squares minimisation method. Otherwise, AR markers are used to guide the AUV.

Park et al. [12] and D. Li et al. [11] were able to obtain the relative two-dimensional position in pixel units of dock to AUV and control the heading direction of the AUV to the dock. Y. Li et al. [13] and N. Palomeras et al. [14] were able to obtain the six-dimensional pose of the AUV, but the computational burden of the former was heavy while the equipment required for the docking station was too complicated for application in turbid water in the case of the latter.

This article focuses on the docking stage, assuming that the AUV has been guided to the lamp field near the docking station using remote guidance technologies. In this study, a fast binocular localisation method is proposed. The proposed scheme uses two packaged CMOS cameras installed below a ship model and three navigation lamps equipped on a testbed docking station. An adaptively weighted OTSU method is presented to extract the lamps more accurately and efficiently, which is described specifically in Section 3. By choosing the mass centres of the extracted lamp objects as the matching feature, the six-dimensional pose of the AUV can be computed with a binocular localisation method at an operation frequency greater than 10 Hz. To address situations in which two cameras cannot capture all the lamps, an efficacious estimation method (D. Li et al. [11]) is applied to obtain the relative two-dimensional position to control the heading direction only.

In the remaining part of this article, Section 2 presents the configuration for the proposed vision guidance system. Section 3 describes the realisation of the proposed system, which includes the processing of the raw images, the binocular vision algorithm, and the control strategy for the ship model. Section 4 presents the experiment results. The final section is a summary of the main conclusions of this study.

## 2. System Configuration

**Figure 1:**
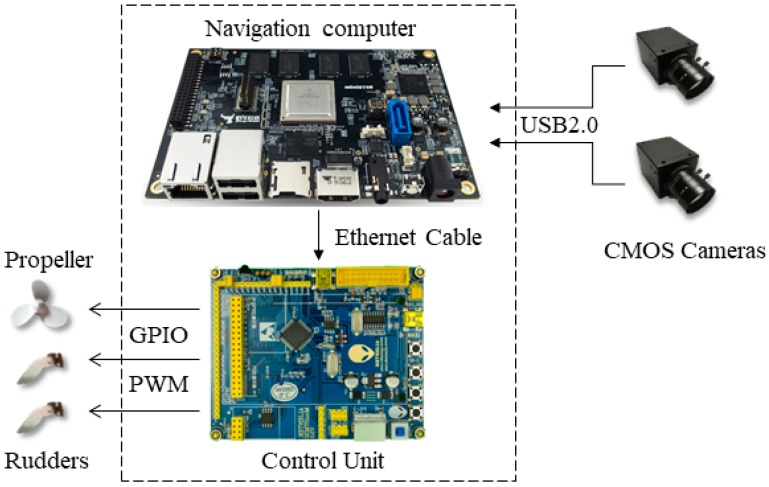
Main components of the test-bed platform.

The platform applied in this article was a ship model. As shown in Figure 1, it contains five main components: two monochromatic Complementary Metal Oxide Semiconductor (CMOS) cameras, two rudders, one propeller, one control unit and one navigation computer. Table 1 presents the details of the equipment specification of the proposed system. We chose a monochromatic camera due to its higher sensitivity to light compared to colour cameras, whose colour filters result in a loss of more than half of the incident light energy [15]. The field angle of the camera is 60 degrees. The CMOS cameras are connected to the navigation computer by Universal Serial Bus (USB) port. The navigation computer is embedded in a Linux operating system and is utilised in processing the images captured by the cameras and then transmitting the visual computation results to the control unit by ethernet. The control unit implements the control strategy (described in Section 3.3) to control the direction and speed of the ship model. To be more specific, it can transmit Pulse Width Modulation (PWM) instructions to the rudders and propeller through General-Purpose Input/Output (GPIO) ports.

**Figure 2:**
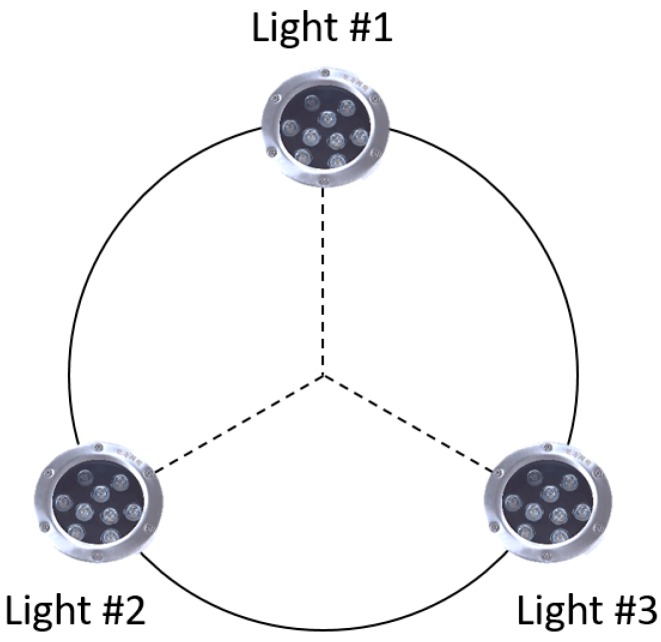
Distribution of the navigation lamps.

Most of the vision guidance systems mount the navigation lamps at the entrance of the docking station as active beacons. In this paper, we also adopt this method. However, instead of a real docking station, we used an aluminium profile model with lamps equipped on it, which operated similarly to the docking station in terms of the terminal docking process. Three common underwater green lamps were symmetrically positioned on the aluminium profile model around the centre of the three lamps, namely, the centre of the docking station (Figure 2). Green lamps were chosen because of the wavelengths at which it is relatively difficult for seawater to either absorb or scatter the light [16].

## 3. Vision Guidance Algorithm

As previously indicated, the vision guidance system primarily contains two cameras installed below the ship model and three active lamps equipped on the simple docking station. This article presents a fast binocular localisation algorithm to determine the relative position and attitude between the AUV and the docking station. A relative localisation algorithm is applied when two cameras cannot capture all three lamps.

**Figure 3:**
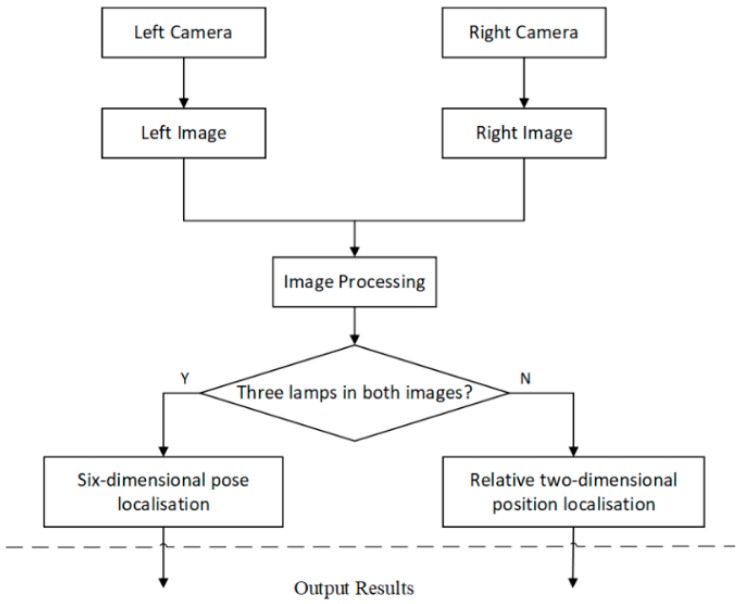
Flow chart of the visual localisation algorithm.

When all three lamps are captured by both cameras, the fast binocular localisation algorithm is able to compute the six-dimensional pose (including three-dimensional position and three-dimensional attitude) of the AUV via a mass-centre matching method. Moreover, a relative localisation method proposed by D. Li et al. [11] is applied when the two cameras cannot capture all the lamps. The combination of these algorithms can improve the reliability of the system without a significant increase in the computational burden. The flow chart of the visual localisation algorithm is shown in Figure 3.

### 3.1. Image Processing

Light is attenuated and scattered during underwater propagation, and hence, images captured in this environment may contain noise and artefacts. In this section, we utilise a median filter to remove image noise and propose a weighted OTSU method to binarise the image and to extract the mass centres of the captured lamps in the binary image.

#### 3.1.1. Image Filtering

**Figure 4:**
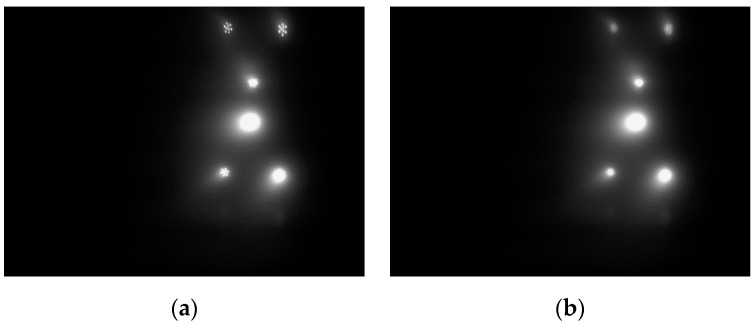
Effect of image filtering: (**a**) the raw image; (**b**) the processed image using the median filter.

An underwater lamp consists of several small LEDs. As such, the captured image of an individual lamp may be identified as several lamps instead of one. A median filter with a 5 × 5 pixel mask was thus adopted to smooth the image and to remove salt-and-pepper noise while still retaining the edge information of the object. Figure 4 shows the raw image and the image acquired after processing using the median filter.

#### 3.1.2. Adaptively Weighted OTSU Method

Before feature matching, the object needs to be extracted from the processed image. It is feasible to segment the foreground object and the background scene through thresholding. In this article, we propose an adaptively weighted OTSU method to obtain the global optimal threshold for the captured image. The traditional OTSU method defines the segmentation threshold as the solution that maximises the between-class variance, which is established as follows:

(1) $$\sigma_{B}^{2}=\omega_{0}{\left(\mu_{0}-\mu_{T}\right)}^{2}+\omega_{1}{\left(\mu_{1}-\mu_{T}\right)}^{2}$$

where *ω*_0_ and *ω*_1_ denote the probability that a pixel is divided into the object and the background under the global threshold T, respectively. The variables *μ*_0_ and *μ*_1_ denote the average grey value of the object zone and the background zone and *μ*_T_ is the average greyscale value of the entire image. The number of pixels divided into the object and the background is denoted as *N*_0_ and *N*_1_, respectively. Assuming *m* and *n* are the width and height of the image in pixel units, respectively, it can be induced from the definition that *ω*_0_ = *N*_0_/(*m* × *n*) and *ω*_1_ = *N*_1_/(*m* × *n*) as well as *N*_0_ + *N*_1_ = *m* × *n*. Therefore, it is obvious to induce that *ω*_0_*μ*_0_ + *ω*_1_*μ*_1_ = *μ*_T_ and *ω*_0_ + *ω*_1_ = 1.

**Figure 5:**
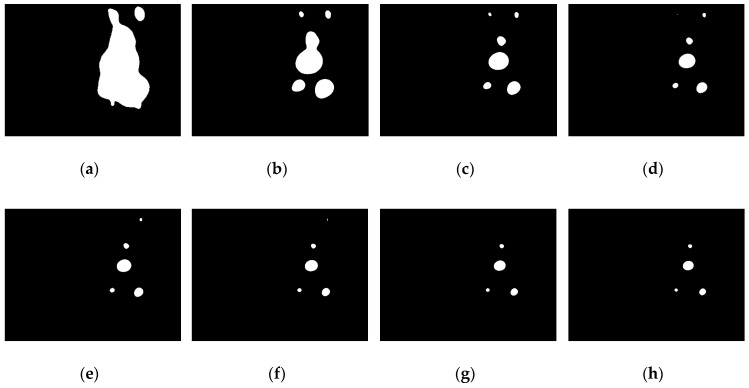

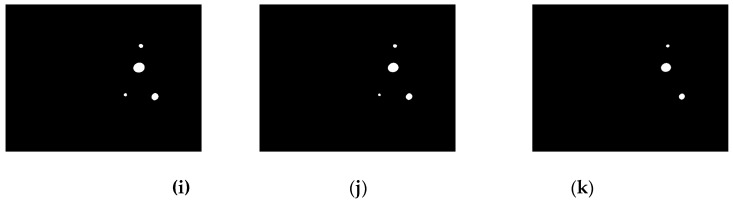
The binary images through (**a**) the original OTSU method and (**b**–**k**) the weighted OTSU method with weight coefficient K from 0.9 to 0 at a step size of 0.1: (**a**) K = 1, T = 48; (**b**) K = 0.9, T = 71; (**c**) K = 0.8, T = 103; (**d**) K = 0.7, T = 128; (**e**) K = 0.6, T = 150; (**f**) K = 0.5, T = 170; (**g**) K = 0.4, T = 188; (**h**) K = 0.3, T = 205; (**i**) K = 0.2, T = 219; (**j**) K = 0.1, T = 233; (**k**) K = 0, T = 245.

After applying the traditional OTSU method, the image shown in Figure 4b becomes the binary image as shown in Figure 5a. The result appears to be non-ideal. The reason for this is that most regions of the image captured underwater are background scene. In other words, the proportion of the pixels with a small grey value (i.e., the background) is much larger than the proportion of the pixels with a large grey value (i.e., the object) in the captured image. Therefore, in order to maximise the between-class variance, the optimal global threshold T calculated by the traditional OTSU method is more likely to be smaller, that is, biases towards the grey value of the background. In this way, some background pixels are segmented as the foreground object, so that the segmented object area is much larger than the real object area and several lamps are mixed together as shown in Figure 5a. This method works well in the study by D. Li et al. [11], because only one light is used for guidance, thereby avoiding the mixing problem. However, in this work, each lamp needs to be extracted individually for the subsequent feature matching process.

To fix the above problem, a weight coefficient is added to increase the proportion of the object in the between-class variance formula. The modified between-class variance is established as follows:

(2) $$\sigma_{B}^{2}{}^{\prime }=\omega_{0}^{K}{\left(\mu_{0}-\mu_{T}\right)}^{2}+\omega_{1}^{2-K}{\left(\mu_{1}-\mu_{T}\right)}^{2}$$

where K is the added weight coefficient. Assuming *ω*_0_ and *ω*_1_ are constant coefficients between 0 and 1, the smaller the value of K is, the larger the value of $\omega_{0}^{K}$ and the smaller the value of $\omega_{1}^{2-K}$ will be. This means that the proportion of the object in the between-class variance will be greater and the extracted objective region will be smaller. Then, in order to maximise the between-class variance, the output threshold of this modified method will bias toward the grey value of the objective region, i.e., the output threshold of this method will be higher than that of the original method. This conclusion is proven in Figure 5.

**Figure 6:**
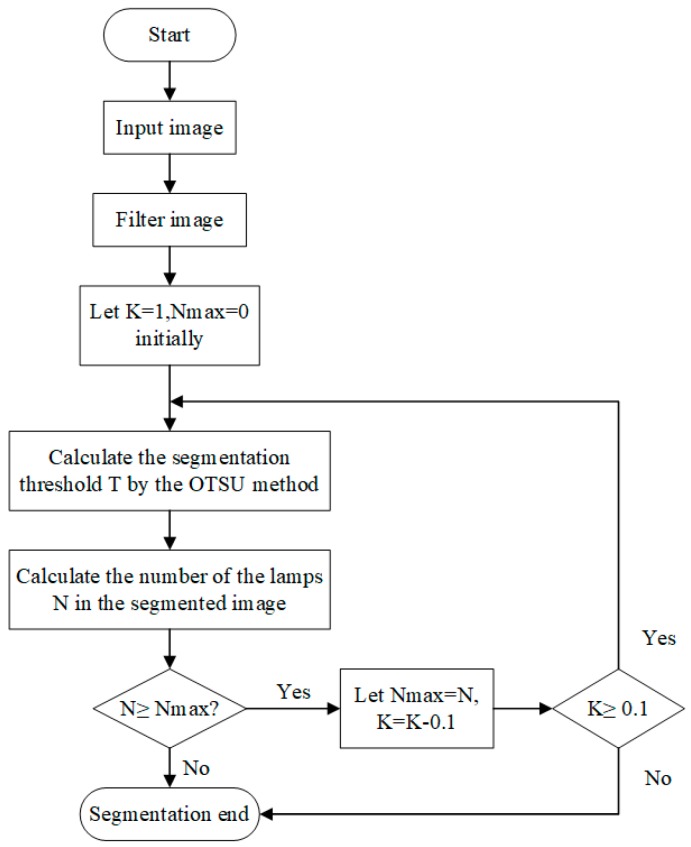
The procedure of the adaptively weighted OTSU method.

The traditional OTSU algorithm is a particular case of the weighted OTSU algorithm when K is equal to 1, as shown in Figure 5a. Figure 5b–k shows the binary images obtained through the weighted OTSU method with different weight coefficients. It can be observed that the smaller the weight coefficient K is, the larger the output global threshold T will be. When K is too small, the output threshold will extremely bias towards the grey value of the objective region. Therefore, the lamps with relatively low grey values cannot be extracted. Taking this condition into consideration, we would like to let K be adaptive according to the number of lamps in the segmented image. The procedures involved in the implementation of the adaptively weighted OTSU method is illustrated in Figure 6. The final weight coefficient K* is selected by maximising the number of lamps in the segmented image N:

(3) $$K^{*}=\underset{0.1\leq K\leq 1}{\mathrm{argmax}}\left\{N\right\}$$

In this method, the extracted lamps are neither mixed nor missed, irrespective of the distance of the AUV from the station.

#### 3.1.3. Image Segmentation

As mentioned in the previous section, threshold segmentation methods (TSMs) are usually used to segment the image into two classes: the objective zone and the background zone. When the boundary threshold T is given, the segmented image can be obtained as follows:

(4) $$g\left(i,j\right)=\{\begin{array}{cc} 255, & f\left(i,j\right)\geq T\left(i,j\right)\\ 0, & f\left(i,j\right)<T\left(i,j\right) \end{array}$$

where f(i,j) and g(i,j) are the grey values of pixel point in the location (i,j) on the processed image and on the binary image, respectively. The pixel point with a grey value greater than or equal to T(i,j) becomes the bright group (grey value equal to 255) while the pixel point with a grey value smaller than T(i,j) becomes the dark group (grey value equal to 0). As such, the greyscale image is divided into the objective zone and the background zone.

Depending on whether the threshold T varies with the location of the pixel point, TSMs can be divided into local TSMs and global TSMs. The threshold of the former method is determined adaptively by the neighbourhood window that is centred on each pixel [17]. In the latter method, the determination of the global threshold is of the most importance. Several studies on vision guidance technology have adopted diverse methods for determining the global threshold. Park et al. [12] adopted a pre-specified threshold value to segment the greyscale image. D. Li et al. [11] applied a traditional OTSU method to obtain the global optimal threshold, which has been commonly used for image segmentation due to its excellent performance. Y. Li et al. [13] used a Mean-Shift algorithm to extract the light source zone. This algorithm is an iterative optimisation approach, which is computationally intensive. The computational burdens of the four aforementioned segmentation methods and the proposed adaptively weighted OTSU approach are illustrated in Table 2. It is evident that the Mean-Shift algorithm takes more than ten times as long as the TSMs and is unsuitable for the real-time processing of images.

**Figure 7:**
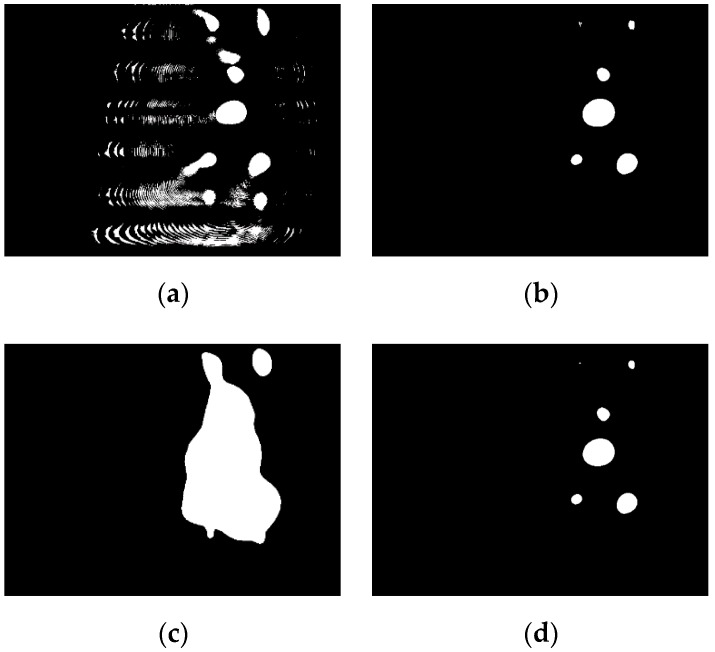
The threshold segmentation results of the four TSM: (**a**) Adaptive local TSM; (**b**) Pre-specified TSM, set T as 125; (**c**) Traditional OTSU TSM, T = 48; (**d**) Adaptively weighted OTSU TSM, T = 128.

**Figure 8:**
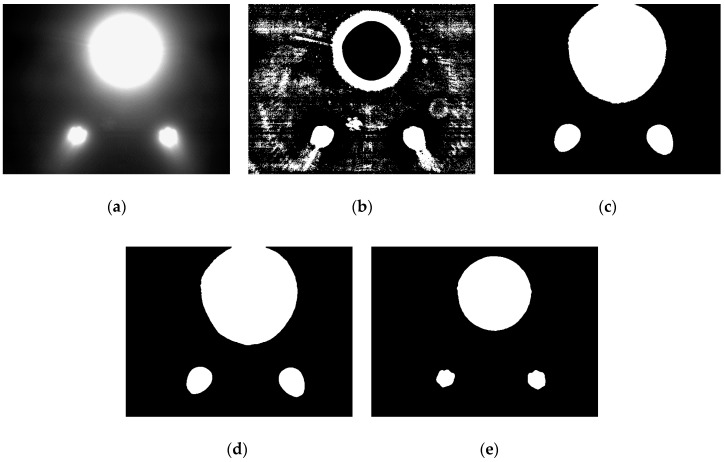
The threshold segmentation results for image captured near the station: (**a**) The original image; (**b**) Adaptive local TSM; (**c**) Pre-specified TSM, set T as 125; (**d**) Traditional OTSU TSM, T = 130; (**e**) Adaptively weighted OTSU TSM, T = 235.

The threshold segmentation results of the four TSMs for the image shown in Figure 4b are illustrated in Figure 7, and the threshold segmentation results for the image captured near the station are shown in Figure 8. It can be concluded that the adaptive local TSM is unsuitable for underwater lamp images due to its high sensitivity to the noise in the image. The pre-specified TSM is the fastest algorithm, but it is inflexible, owing to the brightness of the captured lamps varying with the distance between the AUV and the docking station. For example, when setting the pre-defined threshold as 125, this method appears to be suitable for images captured far from the station (Figure 4b), but it fails to precisely extract the object when the image is captured near the station (Figure 8c). With the traditional OTSU TSM, it is likely to mix up the lamps when the AUV is far from the station, in which cases most regions in the underwater captured images are background (Figure 7c). Moreover, the adaptively weighted OTSU TSM can extract the object precisely without incurring a heavy computational burden, irrespective of the distance between the AUV and the station. Therefore, it outperforms the other four methods.

#### 3.1.4. Feature Extraction

**Figure 9:**
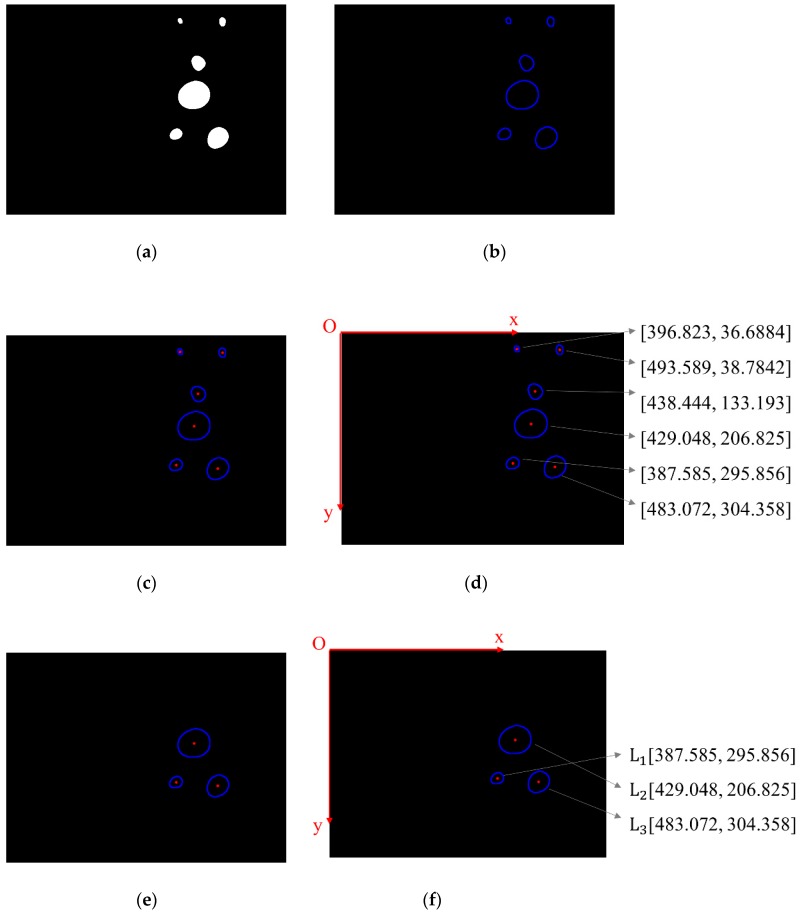
The processes of feature extraction: (**a**) obtain the binary image; (**b**) extract the contours of the lamps; (**c**) obtain the mass centres of each contour; (**d**) sort the mass centres in terms of their y-coordinates; (**e**) remove the reflected lamps; (**f**) sort the mass centres of the lamps in terms of their x-coordinates and mark the three lamps in order.

In this study, we chose the mass centres of the captured lamps as the matching feature of the binocular algorithm. We extracted all the contours of the lamps from the binary image as shown in Figure 9b. Then the coordinates of pixels on each contour were averaged to obtain the coordinate of the mass centre of each contour, i.e., the mass centre of each lamp (Figure 9c). The origin of the pixel coordinate frame is at the top left corner of the image while the positive directions of the x and y axis are respectively point to the left and upper side of the image as shown in Figure 9d.

The mass centres were firstly sorted in terms of their vertical coordinates. To remove the reflected lamps located at the top of the image, we only kept three mass centres that had larger vertical coordinates (Figure 9e). Then we sorted the mass centres of three lamps in terms of their horizontal coordinates and marked the three lamps in order. In this way, we can identify the lamps. When both cameras capture all three lamps, each lamp captured by the left and right camera can be matched in order.

When the roll angle of the AUV exceeds 60°, this sorting method fails. However, this situation rarely occurs. To ensure the normal functional operation of the AUV, IMU (Inertial Measurement Unit), compass, or other sensors will be applied to maintain the variation of the roll angle on a small fluctuation [18]. This is not discussed herein.

### 3.2. Binocular Vision Algorithm

In this study, we apply a parallel binocular vision algorithm to realise the localisation. The position and attitude of the AUV relative to the docking station can be obtained using three co-planar lamps and two parallel cameras.

**Figure 10:**
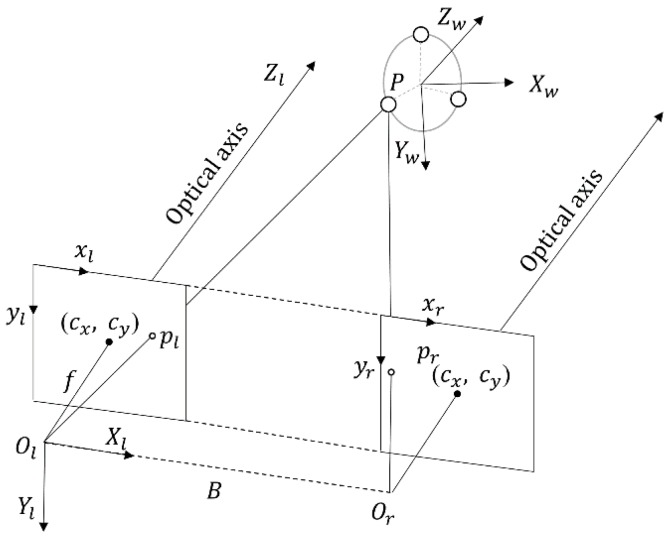
The schematic diagram of the binocular vision algorithm.

Firstly, we adopt the method proposed by Zhang Z. et al. [19] to calibrate the cameras. Using the calibrated internal coefficients and the external coefficients of two cameras, we can rectify the captured images and ensure that the image planes of both cameras are ideally co-planar (Figure 10).

Then, according to the similar triangle theorem, the position of a matching point in the left camera coordinate O_l_-X_l_Y_l_Z_l_ can be computed as:

(5) $$X_{l}=\frac{Z_{l}}{f}\left(x_{l}-c_{x}\right)$$

(6) $$Y_{l}=\frac{Z_{l}}{f}\left(y_{l}-c_{y}\right)$$

(7) $$Z_{l}=\frac{\mathrm{fB}}{x_{l}-x_{r}}$$

where f is the focal length of the camera, B is the baseline length of the two cameras, x_l_ and x_r_ are the horizontal positions of a point in the image coordinate of the left and right camera, respectively, while y_l_ and y_r_ are the vertical positions. *c_x_* and *c_y_* are, respectively, the horizontal and vertical position of the optical centre in the image plane.

**Figure 11:**
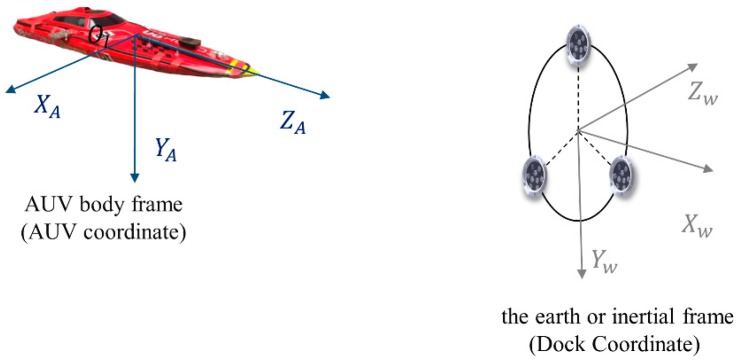
The coordinate frames of the vision guidance system.

The coordinate frames of the vision guidance system in this study are shown in Figure 11. In the AUV body frame, the position of the matching point can be obtained as:

(8) $$X_{A}=X_{l}+\frac{B}{2}=\frac{Z_{l}}{f}\left(x_{l}-c_{x}\right)+\frac{B}{2}$$

(9) $$Y_{A}=Y_{l}=\frac{Z_{l}}{f}\left(y_{l}-c_{y}\right)$$

(10) $$Z_{A}=Z_{l}=\frac{\mathrm{fB}}{x_{l}-x_{r}}$$

The dock coordinate refers to the earth or the inertial frame. The position of the matching points in the AUV coordinate P_A_ can be computed from Equations (8)–(10), while the positions of the three lamps in the dock coordinate P_D_ are determined. Therefore, using the three matching points, the relative translation and rotation of the two coordinates, namely the position and attitude of the AUV relative to the dock, can be computed by the transverse formula:

(11) $$P_{A}={\mathrm{RP}}_{D}+T$$

where T is the translation vector and R is the rotation matrix. Given that the average position of the three lamps is the origin of the dock coordinate, T can be computed as the average position of the three matching points in the AUV coordinate as follows:

(12) $$T=\left[\begin{array}{c} X\\ Y\\ Z \end{array}\right]=P_{\mathrm{aver}\_\mathrm{in}\_\mathrm{AUV}}$$

Then, R can be computed from Equation (11) via the positions of the three matching points in the AUV and dock coordinate. Assuming that ψ, θ and φ are the rotation angles of the AUV coordinate relative to the dock coordinate in the X, Y, and Z direction, R can be described as:

(13) $$R=\left[\begin{array}{ccc} \cos\theta \cos\varphi & \sin\psi \sin\theta \cos\varphi -\cos\psi \cos\varphi & \cos\psi \sin\theta \cos\varphi +\sin\psi \sin\varphi \\ \cos\theta \sin\varphi & \sin\psi \sin\theta \sin\varphi +\cos\psi \cos\varphi & \cos\psi \sin\theta \cos\varphi -\sin\psi \cos\varphi \\ -\sin\theta & \sin\psi \cos\theta & \cos\psi \cos\theta \end{array}\right]$$

Assuming R_ij_ is the element of matrix R in the ith row and the jth column, the attitude of AUV can be obtained from Equation (13):

(14) $$\{\begin{array}{c} \psi =\arctan\frac{R_{32}}{R_{33}}\\ \theta =-\arctan\frac{R_{31}}{\sqrt{R_{11}^{2}+R_{21}^{2}}}\\ \varphi =\arctan\frac{R_{21}}{R_{11}} \end{array}$$

**Figure 12:**
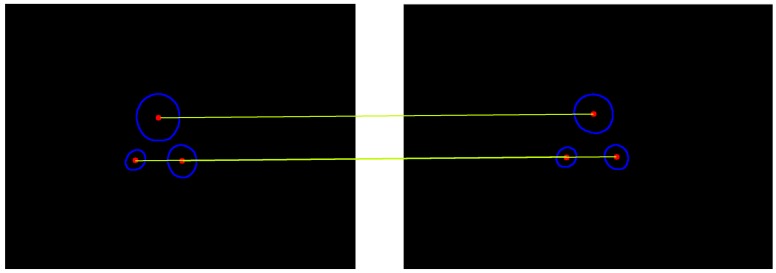
The matching features of the images captured by two cameras.

Hence, the three-dimensional position and three-dimensional attitude of AUV relative to the docking station are deduced. The matching features of the images captured by the two cameras are discussed in Section 3.1 and are shown in Figure 12. With this feature matching method, the operation frequency of the binocular ranging algorithm can exceed 10 Hz and the entire visual process can reach 5 Hz, which satisfies the control requirement.

When both cameras cannot capture all the lamps, we can only calculate the relative distance at the X and Y dimension between the docking station and the AUV. The average coordinates of the mass centres of the captured lamps in the left and right image plane can be computed from the processed image, denoted as (${\bar{x}}_{l}$, ${\bar{y}}_{l}$) and (${\bar{x}}_{r}$, ${\bar{y}}_{r}$), respectively, in pixel units. Then the position of the docking station relative to the AUV ($\bar{x}$, $\bar{y}$) in pixel units can be computed as follows:

(15) $$\bar{x}=\frac{x_{l}+x_{r}}{2}-\frac{m}{2}$$

(16) $$\bar{y}=\frac{y_{l}+y_{r}}{2}-\frac{n}{2}$$

### 3.3. Control Strategy

The motion control system for AUV consists of two independent parts, namely, the control strategies for the horizontal and vertical plane [20]. The control schemes for motion in two planes are similar [11]. To avoid redundancy, we only focus on the tracking control strategy on one plane and the other plane can be derived in a similar manner. Furthermore, during the docking stage in a real scenario, the AUV tends to sail at a setting depth and to move on the horizontal two-dimensional plane. Hence, in this study, we only discuss the two-dimensional motion control on the horizontal plane and carry out the experiment in the same way. The classical Proportion Integration Differentiation (PID) control strategy is used to control the yaw angle of the ship model on the horizontal plane.

When both cameras are able to capture all three lamps, we can obtain the three-dimensional position and attitude of the AUV relative to the docking station from the binocular vision algorithm. From the obtained three-dimensional position, the horizontal yaw angle of the ship model relative to the docking station can be obtained as follows:

(17) $$\theta =\arctan\frac{X_{A}}{Z_{A}}$$

where X_A_ and Z_A_ denote the position of the docking station in the X and Z direction of the AUV coordinate, respectively.

**Figure 13:**
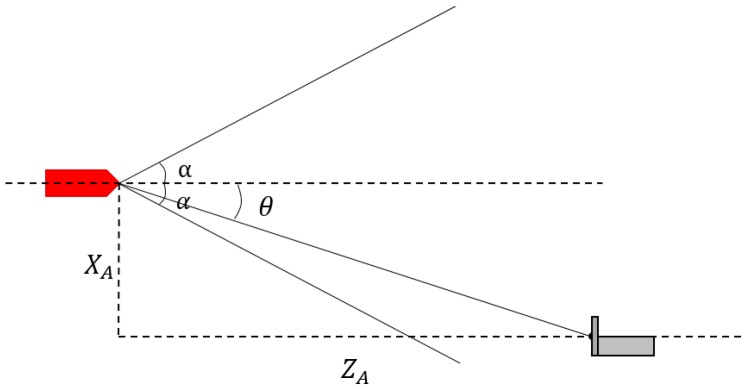
Schematic of AUV on the horizontal plane.

When the two cameras cannot capture all three lamps, we can only obtain the relative position of the docking station relative to the ship model in pixel units ($\bar{x}$, $\bar{y}$). In such cases, Equation (17) is not applicable. Assuming that the field angle of the camera is α, we can obtain the following equation via the geometric relationship and the camera model shown in Figure 13:

(18) $$\frac{k\cdot m/2}{Z_{A}}=\tan\frac{\alpha }{2}$$

(19) $$\frac{k\cdot \bar{x}}{Z_{A}}=\tan\theta$$

where k is the proportional coefficient.

Therefore, the horizontal yaw angle of the ship model can be derived as:

(20) $$\theta =\arctan\left(\frac{2\bar{x}}{m}\times \tan\frac{\alpha }{2}\right)$$

**Figure 14:**
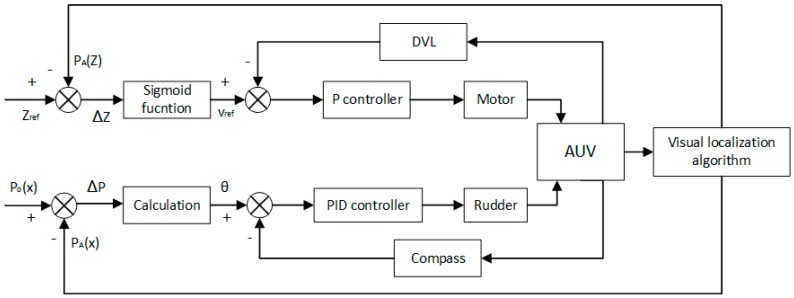
The schematic diagram of the control strategy on the horizontal plane.

The PID control scheme is illustrated in Figure 14. The position of the docking station in the AUV coordinate P_A_ can be obtained by the proposed vision localisation algorithm, while P_O_ denotes the origin of the AUV coordinate. The deviation between P_A_ and P_O_, denoted as ΔP, is equal to P_A_. Thereby, the yaw angle θ can be calculated by Equations (17) and (20). The compass can measure the angle of the centre line of the AUV deviated from the centre line of the docking station θ_dock_. The deviation between θ and θ_dock_, denoted as Δθ, serves as the input of the controller, the coefficients of which are determined by trial and error.

Moreover, it is possible for AUV to obtain its six-dimensional pose relative to the docking station when the binocular vision algorithm works. Therefore, except for the heading direction, the AUV can control its speed as well. When the target position in Z direction P_A_(z) is larger than the reference Z-distance Z_ref_, which is set as 5 m, it is suggested that the AUV moves at a relatively high velocity. When Z_A_ is smaller than Z_ref_, it is suggested that the AUV moves at a relatively low velocity to avoid strong collision with the docking station. The relationship between the deviation ∆Z and the reference velocity v_ref_ is established via the sigmoid function:

(21) $$v_{\mathrm{ref}}=\frac{K_{v}}{1+e^{\Delta Z}}+v_{0}$$

where v_0_ is the minimum velocity of the AUV and K_v_ + v_0_ is the maximum velocity. K_v_ is set as v_0_ so that the maximum velocity is twice the minimum velocity.

In conditions where the two cameras cannot capture all three lamps, the AUV is either within short range of the docking station or has a large deviation from the docking station. In this case, it is desirable that the AUV moves slowly, and hence we let Z_A_ be equal to 0 m. The control strategy scheme on the horizontal plane is shown in Figure 14.

## 4. Pool Experiment

The aim of this research is to develop a method for a platform-based docking station such that AUVs only need to be laid on the platform without the requirement of extremely high precision. In this case, only the three-dimensional position is required to control the AUV to complete its docking process. We initially tested the localisation accuracy of the distance in the heading direction, namely the localisation accuracy of the proposed binocular vision system. A verification experiment was then performed in the laboratory pool to validate the feasibility of the entire system.

### 4.1. Experiment Platform

**Figure 15:**
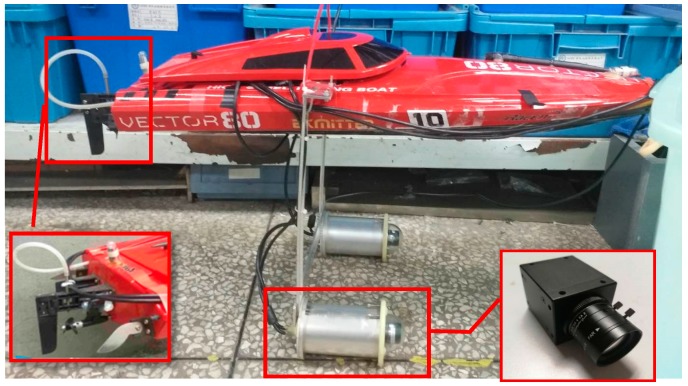
The ship model used in this experiment.

The experiment aims to verify the feasibility of the presented localisation method in which the impact of the experimental object size turns out to be small. The ship model used in this experiment is shown in Figure 15. The binocular cameras are installed about twenty centimetres below the ship model, while the navigation computer and control unit are contained in the inner cavity. The motor and the rudders are located at its tail.

**Figure 16:**
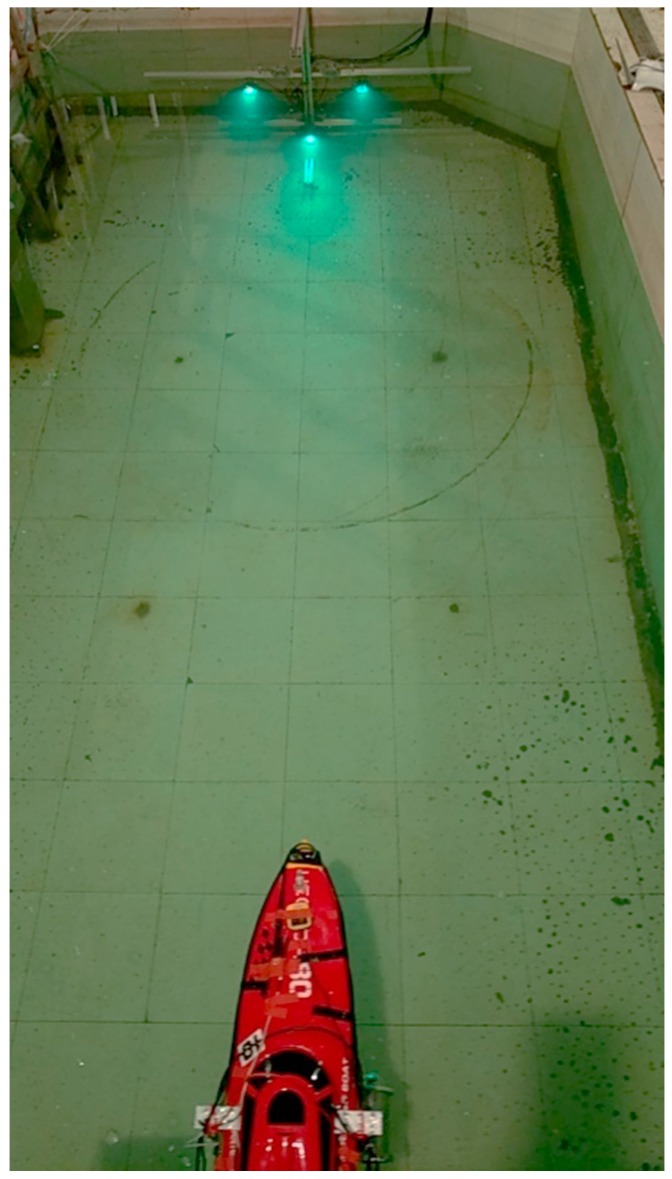
The general lab pool for experiment.

The experiment was conducted in an indoor laboratory pool with dimensions of 4.2 × 2.4 × 1.2 m^3^, as shown in Figure 16. A simple docking station with three navigation lamps was positioned on one side of the water pool. Throughout the entire experiment, the ship model moves automatically, without connecting to any wire cables. The starting and stopping of the ship are controlled via Wi-Fi signals, while the movement is autonomous. The cameras installed below the ship model capture images of the lamps and the navigation computer calculates the three-dimensional position of the ship model relative to the docking station. This data is input into the control unit to control the yaw angle of the ship on the horizontal plane, and to finally lead it to the docking station. The positions are then recorded in the navigation computer.

### 4.2. Test of Localisation Accuracy

To validate the accuracy of the binocular vision algorithm with the proposed feature matching method, we performed a test on the localisation accuracy of the distances in the heading direction at fixed points. The distance in the heading direction instead of the deviation direction was selected, because the former had a larger test range in the pool.

The binocular cameras were positioned at fixed points underwater within a range of 1.2 m to 3.6 m away from the simple docking station. The computed distances from the binocular vision algorithm are compared with the actual distances measured by the tapeline, which is illustrated in Table 3. It can be obtained that the average localisation error is approximately 5 cm and the average relative localisation error is close to 2%, which satisfies the requirement for the tracking control of the AUV.

### 4.3. Docking Experiment

**Figure 17:**
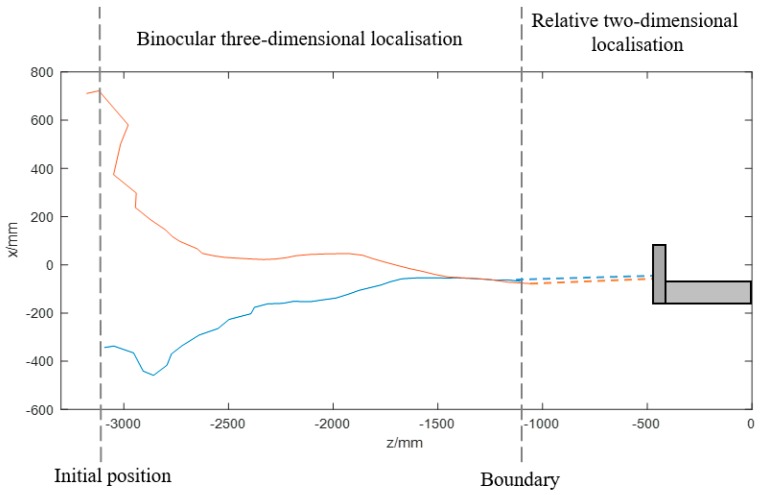
Horizontal trajectories of the ship model at different initial lateral deviations: the blue line illustrates the motion situation on the horizontal plane when the ship model starts at the right side of the docking station; the red line illustrates the situation when the ship model starts at the left side.

**Figure 18:**
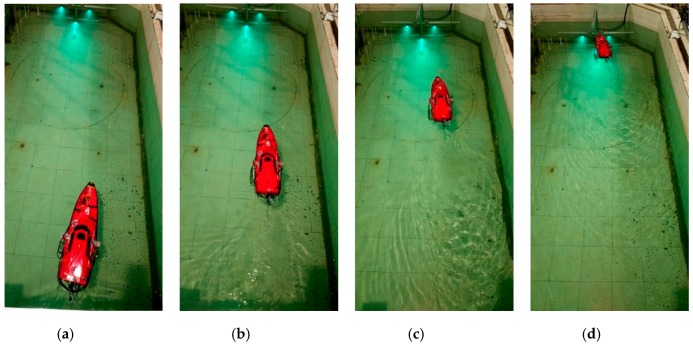
Several photographs acquired during the docking process when the ship model starts at the right side of the docking station: The ship model (**a**) sets off from the initial position on the right; (**b**) turns left to the target; (**c**) moves towards it; (**d**) achieves the docking station successfully.

**Figure 19:**
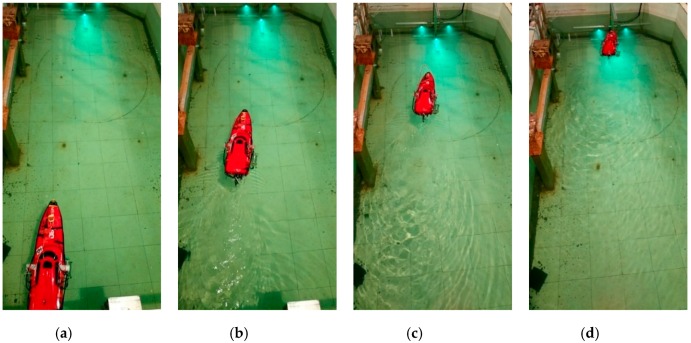
Several photographs acquired during the docking process when the ship model starts at the left side of the docking station: The ship model (**a**) sets off from the initial position on the left; (**b**) turns right to the target; (**c**) moves towards it; (**d**) achieves the docking station successfully.

To verify the feasibility of the entire visual system, the ship model was positioned approximately 3.1 m from the docking station at different initial lateral deviations. The experiment results are shown in Figure 17, Figure 18 and Figure 19.

The blue line in Figure 17 illustrates the motion displacement computed by the binocular vision algorithm on the horizontal plane when the ship model starts at the left side of the docking station. Its path is in reverse at the beginning, because it is in a slant direction. It takes time for the ship model to move towards the docking station by adjusting its yaw angles at a small P coefficient, as determined by the control strategy. The red line illustrates the condition in which the ship model starts at the right side of the docking station.

When the ship is approximately 1.1 m away from the docking station, the cameras cannot capture all the lamps, so the ship cannot obtain its three-dimensional position relative to the docking station. In such circumstances, the ship model keeps moving to the docking station according to the computed relative two-dimensional position in pixel units. Figure 18 and Figure 19 show that the ship model can successfully achieve docking irrespective of its initial position relative to the docking station.

## 5. Conclusions

This study presents a fast binocular localisation method in combination with a relative pose estimation method for the instance when two cameras cannot capture all the lamps. A detailed description of the system configuration is proposed. Through image processing, including image filtering, image segmentation using an adaptively weighted OTSU method and feature extraction, the mass centres of the lamps are obtained as the matching features of the binocular algorithm. A control strategy based on this vision algorithm is then provided. The test at fixed points shows that the relative localisation error in the heading direction within 3.6 m is approximately 2%, which satisfies the requirements for tracking control. A verification experiment was conducted in the laboratory pool using a ship model to evaluate the feasibility of the entire system. The ship model can achieve docking no matter whether it starts at the right side or the left side of the docking station.

Compared with the other vision guidance systems mentioned in the introduction, the vision guidance system presented in this article has the following advantages. Firstly, an adaptively weighted OTSU method is proposed to segment the captured image with good performance and a relatively low cost of computation compared with other TSMs. Secondly, the frequency of the proposed binocular localisation method can reach up to 10 Hz, and the entire algorithm can achieve 5 Hz. Furthermore, the vision algorithm includes a relative pose estimation method in the case when there are one or more lamps out of the viewing field of the cameras. The vision guidance algorithm will work even if only one of the cameras captures lamps, greatly extending its working range.

However, some defects still exist in this article and can be optimised in the future. For example, the pool experiment conducted in this article uses a model ship to validate the proposed algorithm. In future work, experiments using a full-sized AUV will be conducted in a deep-water area to evaluate the effectiveness of the proposed system in an actual sea environment. The motion in the vertical plane will be taken into consideration. Moreover, to make this vision guidance algorithm more practical, a dead reckoning algorithm could be used as an assisted algorithm for circumstances when the target is lost from the sight of both cameras during the docking stage. In addition, a laser source may be considered in the future to extend the effective range of the vision guidance system.

## Figures and Tables

**Table 1:** Specifications of the main equipment.

Item	Model	Position	Number
Monochromatic CMOS Camera	MV-UB130T	Below the ship	2
Navigation Computer	MINI5728	Inside the ship	1
Control Unit	STM32F103ZET6	Inside the ship	1
Underwater green lamp	SF-SXD-001	At the entrance of the docking station	3

**Table 2:** Computational costs of different segmentation method.

Segmentation Method	Computational Time/ms
Adaptive local TSM [17]	0.51777
Pre-specified TSM [12]	0.01926
Traditional OTSU TSM [11]	0.63905
Mean-Shift algorithm [13]	183.239
Adaptively weighted OTSU TSM	17.641

**Table 3:** The test results of the distances in the heading direction.

The Measured Distance (mm)	The Computed Distance (mm)	The Absolute Error (mm)	The Relative Error (%)
1200	1188.9	11.1	0.92
1500	1512.18	12.18	0.81
1800	1795.55	4.45	0.25
2100	2040.12	59.88	2.85
2400	2361.62	38.38	1.60
2700	2575.64	124.36	4.61
3000	3088.04	88.04	2.93
3300	3238.22	61.78	1.87
3600	3501.35	98.65	2.74

## References

[1] Eichhorn M., Ament C., Jacobi M., Pfuetzenreuter T., Karimanzira D., Bley K., Wehde H. (2018). Modular AUV System with Integrated Real-Time Water Quality Analysis. Sensors.

[2] Yang C., Lin M., Li D. (2018). Improving Steady and Starting Characteristics of Wireless Charging for an AUV Docking System. IEEE J. Ocean. Eng..

[3] Lin M., Li D., Yang C. (2017). Design of an ICPT system for battery charging applied to underwater docking systems. Ocean Eng..

[4] Mcewen R.S., Hobson B.W., Mcbride L., Bellingham J.G. (2009). Docking Control System for a 54-cm-Diameter (21-in) AUV. IEEE J. Ocean. Eng..

[5] Allen B., Austin T., Forrester N., Goldsborough R., Stokey R. (2006). Autonomous Docking Demonstrations with Enhanced REMUS Technology. Oceans.

[6] Vallicrosa G., Ridao P., Ribas D., Palomer A. Active Range-Only beacon localization for AUV homing. Proceedings of the 2014 IEEE/RSJ International Conference on Intelligent Robots and Systems.

[7] Feezor M.D., Sorrell F.Y., Blankinship P.R., Bellingham J.G. (2001). Autonomous underwater vehicle homing/docking via electromagnetic guidance. IEEE J. Ocean. Eng..

[8] Vandavasi B.N., Arunachalam U., Narayanaswamy V., Raju R., Vittal D.P., Muthiah R., Gidugu A.R. (2018). Concept and testing of an electromagnetic homing guidance system for autonomous underwater vehicles. Appl. Ocean Res..

[9] Peng S., Liu J., Wu J., Li C., Liu B., Cai W., Yu H. (2019). A Low-Cost Electromagnetic Docking Guidance System for Micro Autonomous Underwater Vehicles. Sensors.

[10] Fan S., Liu C., Li B., Xu Y., Xu W. (2018). AUV docking based on USBL navigation and vision guidance. J. Mar. Sci. Technol..

[11] Li D., Zhang T., Yang C. (2016). Terminal Underwater Docking of an Autonomous Underwater Vehicle Using One Camera and One Light. Mar. Technol. Soc. J..

[12] Park J.Y., Jun B.H., Lee P.M., Oh J. (2009). Experiments on vision guided docking of an autonomous underwater vehicle using one camera. Ocean Eng..

[13] Li Y., Jiang Y., Cao J. (2015). AUV docking experiments based on vision positioning using two cameras. Ocean Eng..

[14] Palomeras N., Vallicrosa G., Mallios A., Bosch J., Vidal E., Hurtos N., Carreras M., Ridao P. (2018). AUV homing and docking for remote operations. Ocean Eng..

[15] Adams J., Parulski K., Spaulding K. (1998). Color processing in digital cameras. IEEE Micro..

[16] Baiden G., Bissiri Y., Masoti A. (2009). Paving the way for future underwater omnidirectional wireless optical communication systems. Ocean Eng..

[17] Jain A.K. (1989). Fundamentals of Digital Image Processing.

[18] Paull L., Saeedi S., Seto M., Li H. (2014). AUV Navigation and Localization: A Review. IEEE J. Ocean. Eng..

[19] Zhang Z. (2000). A Flexible New Technique for Camera Calibration. Tpami.

[20] Fossen T.I. (1994). Guidance and Control of Ocean Vehicles.

